# Supplementary material for: IL-3-Induced Immediate Expression of c-fos and c-jun Is Modulated by the IKK2-JNK Axis
Source: Cells. 2022 Apr 25;11(9):1451. doi: 10.3390/cells11091451 (PMC9105775; doi:10.3390/cells11091451)
Supplement: Supplementary file 1 [file cells-11-01451-s001.zip › cells-1701452-supplementary.pdf]

Supplementary Materials

IL-3-induced immediate expression of c-*fos* and c-*jun* is modulated by the IKK2-JNK axis

Hirotaka Fujita, Toshitsugu Fujita * and Hodaka Fujii

Department of Biochemistry and Genome Biology, Hirosaki University Graduate School of Medicine, 5 Zaifu-cho, Hirosaki 036-8562, Aomori, Japan

* Correspondence: toshitsugu.fujita@hirosaki-u.ac.jp; Tel: +81-(0)172-39-5019

Table of Contents

Table S1. Primers used in this study.

Figure S1. Expression levels of c-*fos* and c-*myc* 0 and 30 minutes after IL-3 stimulation in 32D cells in the presence of IKK-16.

Figure S2. Effects of IKK2 KO on c-*fos* and c-*jun* expression.

Figure S3. IL-3-dependent induction of IEGs transcription.

Figure S4. IL-3-induced phosphorylation of IKK1 and IKK2.

Figure S5. Recovery of c-*fos* and c-*jun* expression in IKK1 KO cells by exogenous expression of FLAG-IKK1.

Figure S6. Expression levels of c-*fos*, c-*jun*, and c-*myc* 0 and 20 minutes after IL-3 stimulation in the presence of JNK-IN-8.

Figure S7. Full immunoblot images for the cropped images presented in the main and supplementary figures.

Table S1. Primers used in this study

Number	Name	Sequence (5' → 3')	Experiments
26773	m18SrRNA-F2	CTTAGAGGGACAAGTGGCG	Quantitative RT-PCR of 18S rRNA
26774	m18SrRNA-R2	ACGCTGAGCCAGTCAGTGTA	Quantitative RT-PCR of 18S rRNA
28146	cMyc-F	CCCTAGTGCTGCATGAGGAGACAC	Quantitative RT-PCR of c-*myc* mRNA
28147	cMyc-R	CCACAGACACCACATCAATTTCTTCC	Quantitative RT-PCR of c-*myc* mRNA
28568	c-jun-qPCR-F	AAGGAAGCTGGAGCGGATCG	Quantitative RT-PCR of c-*jun* mRNA
28569	c-jun-qPCR-R	ATGCCAGCTCGGAGTTTTGC	Quantitative RT-PCR of c-*jun* mRNA
28570	c-fos-qPCR-F	GAAGACCGTGTCAGGAGGCA	Quantitative RT-PCR of c-*fos* mRNA
28571	c-fos-qPCR-R	CTCCTCCGATTCCGGCACTT	Quantitative RT-PCR of c-*fos* mRNA
28665	mIKK1-KO-sense	CACCGCGGCTTGGCACCGGCGGTTT	Construction of sgRNA plasmid for IKK1 KO
28666	mIKK1-KO-antisense	AAACAAACCGCCGGTGCCAAGCCGC	Construction of sgRNA plasmid for IKK1 KO
28715	mIKK2-KO-sense	CACCGTGGGAAATGAAAGAACGCCT	Construction of sgRNA plasmid for IKK2 KO
28716	mIKK2-KO-antisense	AAACAGGCGTTCTTTCATTTCCCAC	Construction of sgRNA plasmid for IKK2 KO
28840	mIKK1-InFusion-F	CGCCGGAATTAGATCCAGTGTGCTG GAAACCATGGACTACAAGG	Construction of retroviral plasmid encoding FLAG-IKK1
28841	mIKK1-InFusion-R	CTACCCGGTAGAATTATTCTGCTAAC CAACTCCAATCAAGAC	Construction of retroviral plasmid encoding FLAG-IKK1

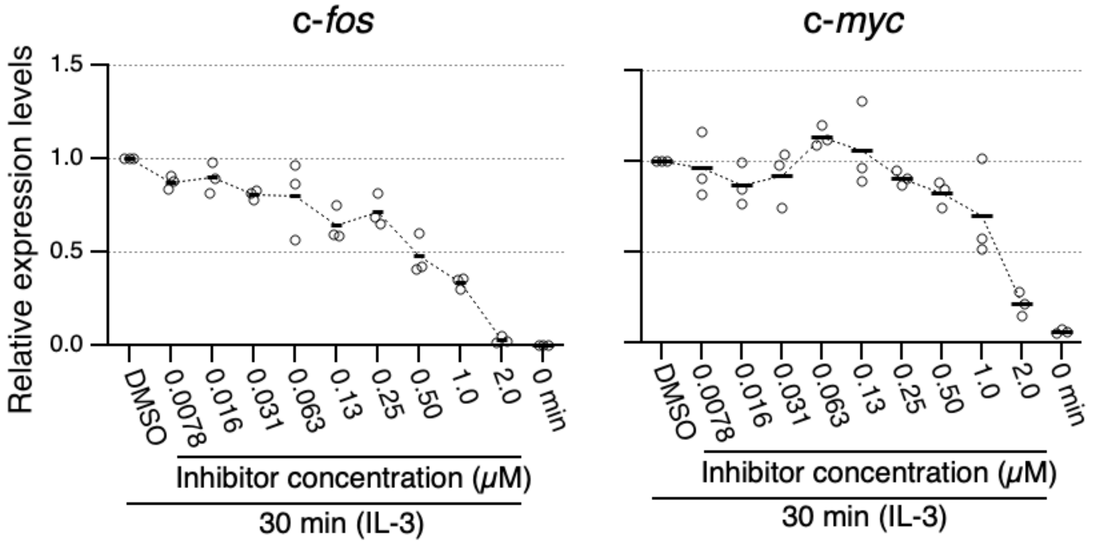

Figure S1. Expression levels of c-*fos* and c-*myc* 0 and 30 minutes after IL-3 stimulation in 32D cells in the presence of IKK-16. 32D cells were pre-incubated with IKK-16 (7.8 nM–2.0 μM) or DMSO for 6 hours and stimulated with IL-3. Each dot represents the result from independent experiments (n = 3 each). Means are indicated by black bars.

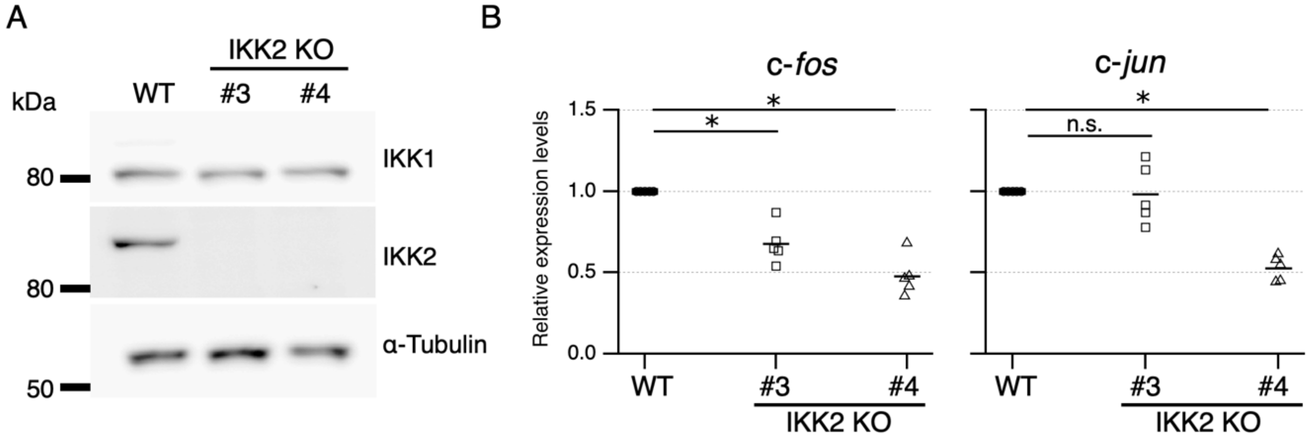

Figure S2. Effects of IKK2 KO on c-*fos* and c-*jun* expression. (**A**) Levels of IKK1 and IKK2 in Ba/F3 parental (WT) and CRISPR/Cas9-mediated IKK2 KO cells. Immunoblot analysis was performed with whole-cell lysates using the indicated Abs. (**B** and **C**) Relative levels of expression of c-*fos* (**B**) and c-*jun* (**C**) mRNAs in Ba/F3 parental and IKK2 KO cells. Expression levels of c-*fos* and c-*jun* mRNA were measured 20 and 40 minutes, respectively, after IL-3 stimulation. *, $p < 0.05$; n.s., not significant, as assessed by the Kruskal–Wallis test with the Steel–Dwass test. Each dot represents the result from independent experiments (n = 5). Means are indicated by bars.

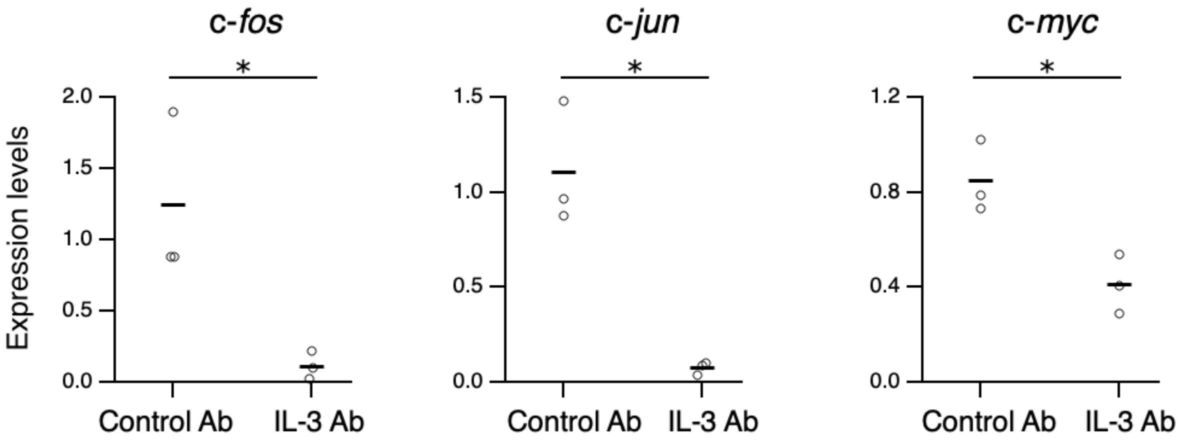

Figure S3. IL-3-dependent induction of IEGs transcription. Ba/F3 cells were starved of IL-3 for 6 hours and re-stimulated with IL-3 pre-incubated with anti-IL3 neutralizing Ab (IL-3 Ab) or control IgG (Control Ab). Total RNA prepared from stimulated cells 20 minutes after stimulation were subjected to RT-qPCR to measure the levels of expression of c-*fos*, c-*jun*, and c-*myc* mRNAs. *, $p < 0.05$, as assessed by Student's *t*-tests for comparisons between IL-3 Ab and control Ab. Each dot represents the result from independent experiments (n = 3). Means are indicated by bars.

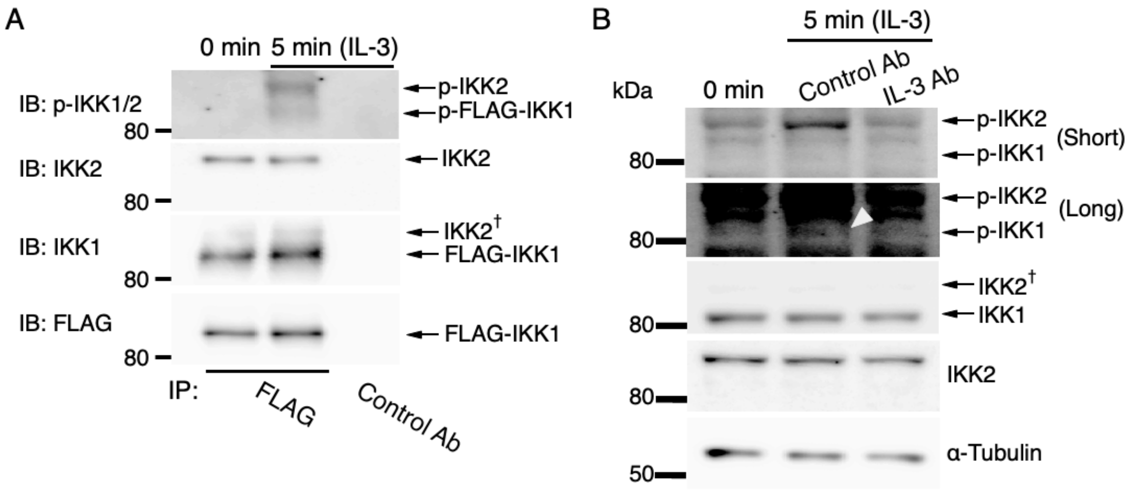

Figure S4. IL-3-induced phosphorylation of IKK1 and IKK2. (**A**) Phosphorylation of immunoprecipitated FLAG-IKK1. IKK1 KO Ba/F3 cells stably expressing FLAG-tagged IKK1 (FLAG-IKK1#1, see Fig. S5) were stimulated with IL-3 after IL-3 deprivation for 6 hours. Immunoprecipitants obtained using anti-FLAG Ab were immunoblotted using the indicated Abs. A dagger denotes the detection of IKK2 by potential cross-reactivity of the anti-IKK1 Ab. (**B**) IL-3-dependent phosphorylation of IKKs. Ba/F3 cells were starved of IL-3 for 6 hours and incubated with IL-3 that had been pre-incubated with anti-IL3 neutralizing Ab (IL-3 Ab) or control IgG (Control Ab). Long and short exposures to detect phosphorylated IKK1/2 are shown. An arrowhead indicates the band corresponding to phosphorylated IKK1.

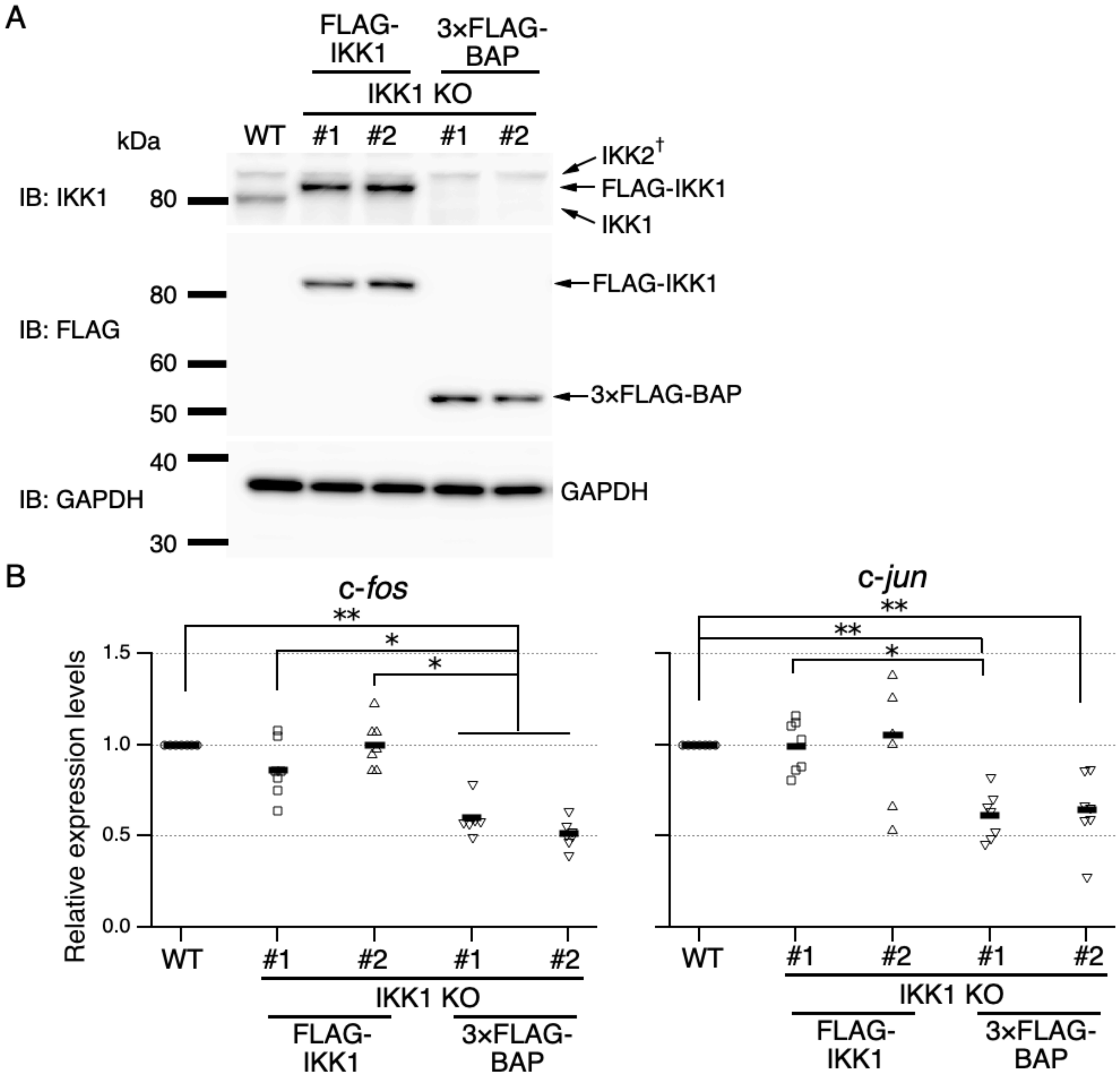

Figure S5. Recovery of c-*fos* and c-*jun* expression in IKK1 KO cells by exogenous expression of FLAG-IKK1. (**A**) Expression levels of endogenous and exogenous IKK1 in Ba/F3 parental (WT) and IKK1 KO cells retrovirally transduced with FLAG-IKK1 or 3×FLAG-BAP. Immunoblot analysis was performed with whole-cell lysates using the indicated Abs. (**B** and **C**) Relative expression levels of c-*fos* (**B**) and c-*jun* (**C**) mRNAs in WT and IKK1 KO cells expressing FLAG-IKK1 or 3×FLAG-BAP. Expression levels of c-*fos* and c-*jun* mRNA were measured 20 and 40 minutes, respectively, after IL-3 stimulation. *, $p < 0.05$; **, $p < 0.01$; n.s., not significant, as assessed by the Kruskal–Wallis test with the Steel–Dwass test. Each dot represents the result from independent experiments (n = 7). Means are indicated by bars.

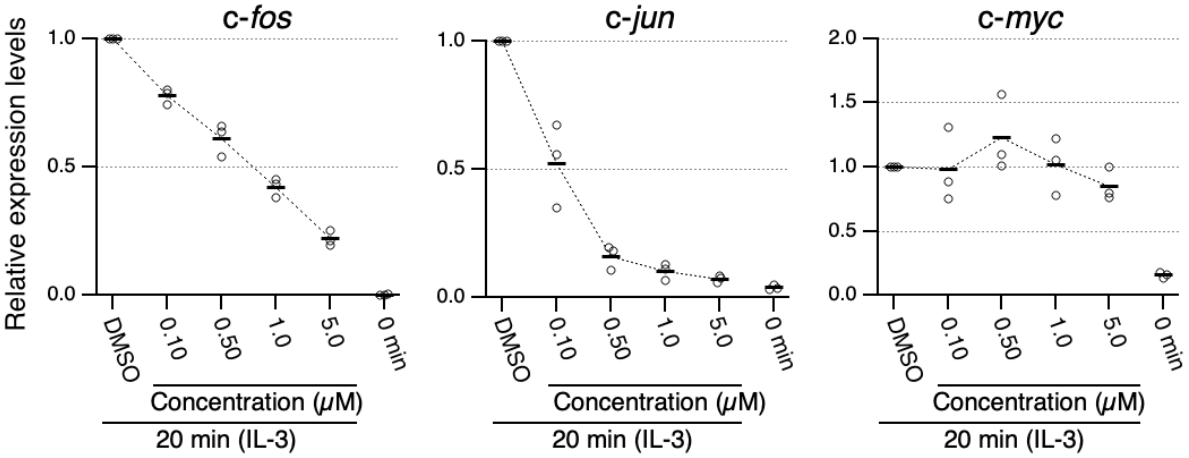

Figure. S6. Expression levels of c-*fos*, c-*jun*, and c-*myc* 0 and 20 minutes after IL-3 stimulation in the presence of JNK-IN-8. Ba/F3 cells were pre-incubated with JNK-IN-8 (0.10–5.0 μM) or DMSO for 6 hours, and stimulated with IL-3. Each dot represents the result from independent experiments (n = 3 each). Means are indicated by black bars.

Fig. S7 (from here)

Full western blots
Figure 2A

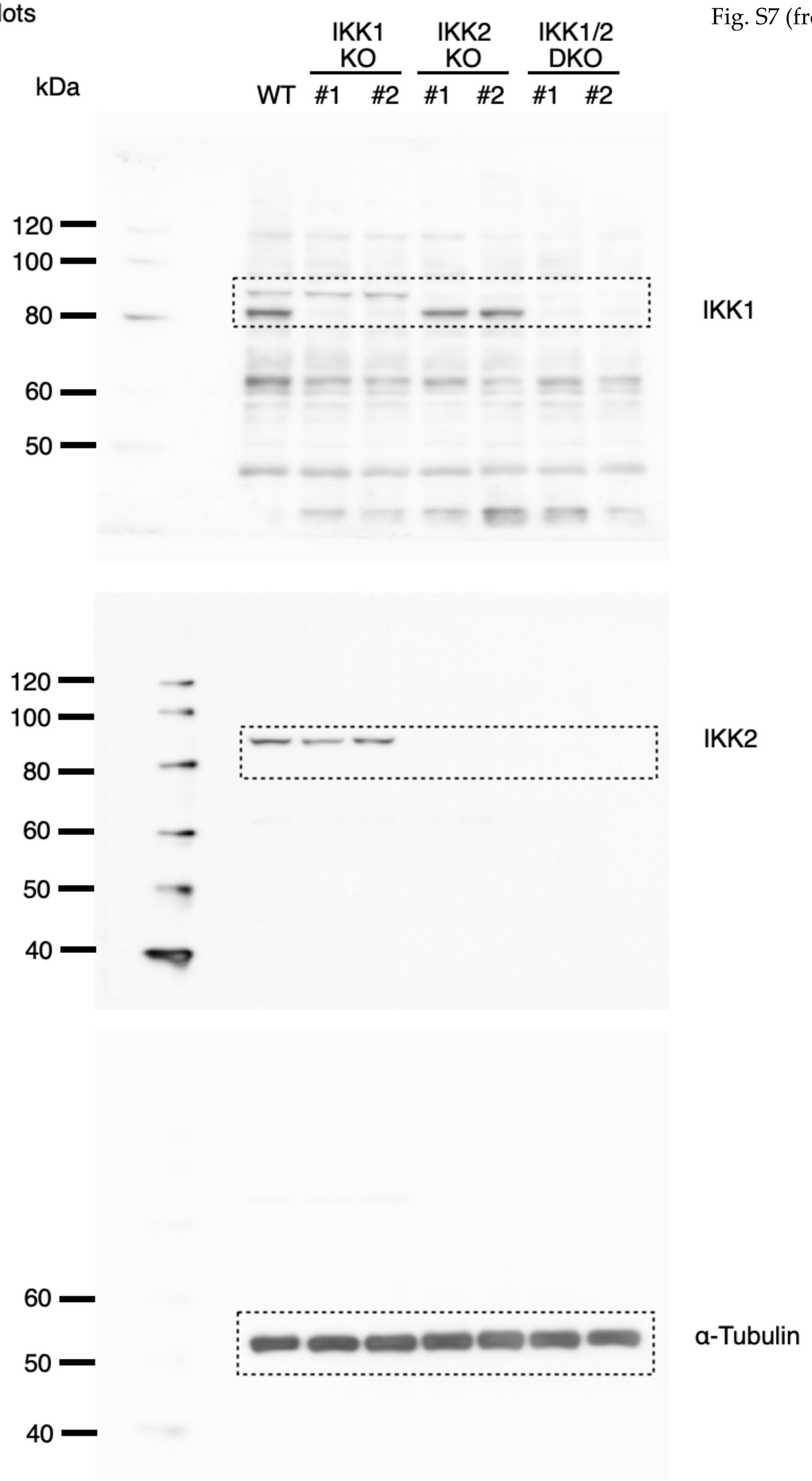

Full western blots
Figure 3A

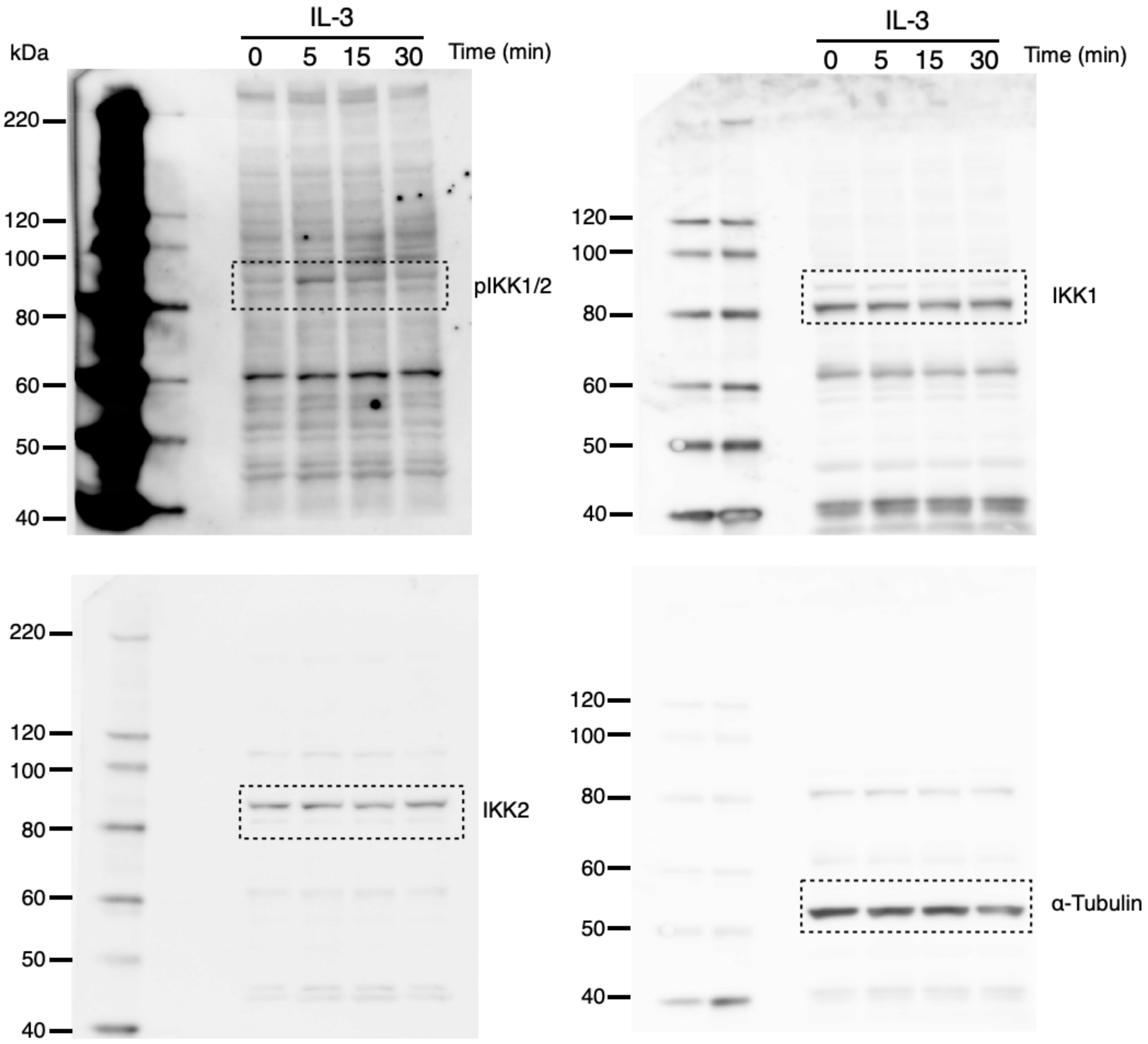

Full western blots
Figure 3B

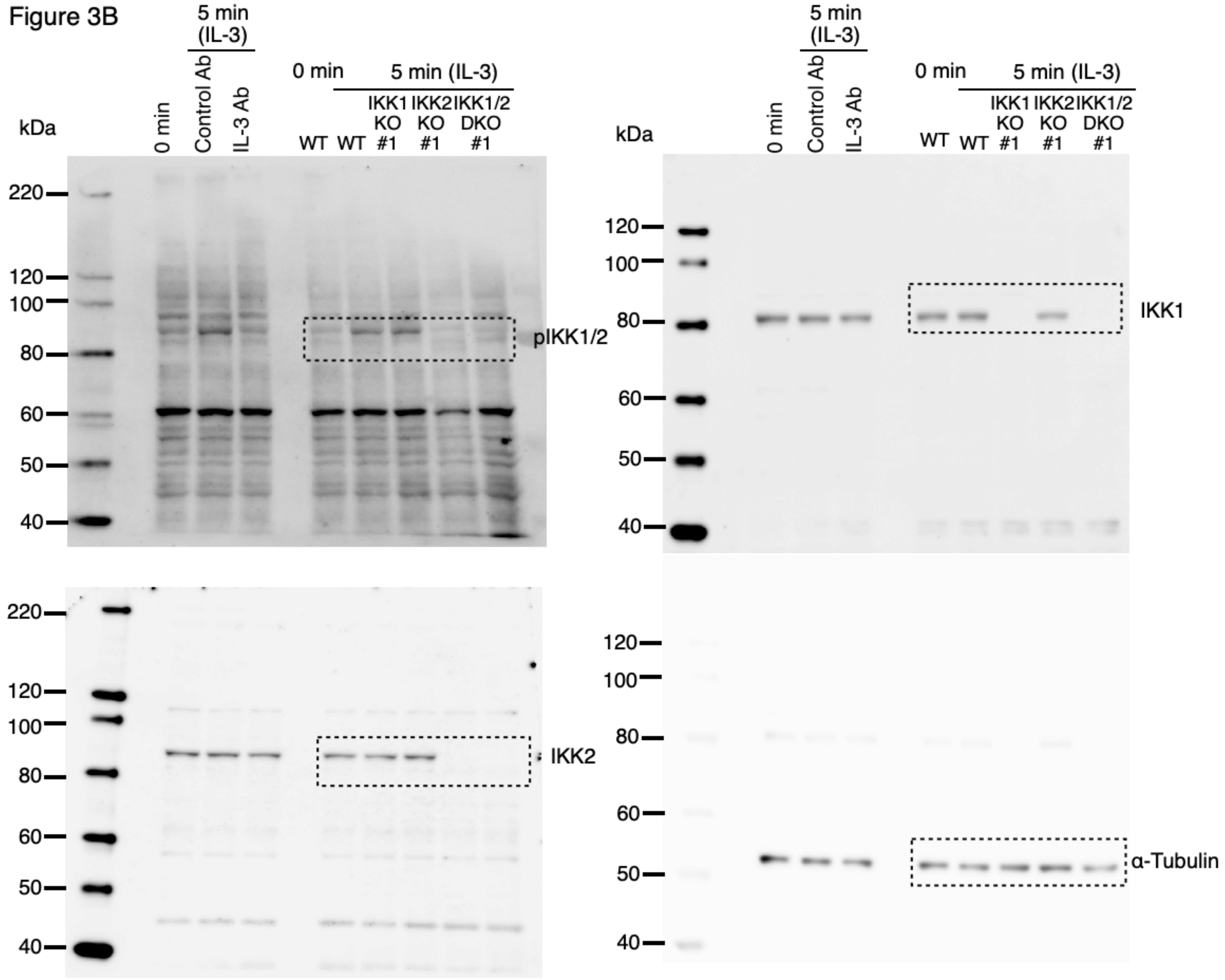

Full western blots
Fig 3C

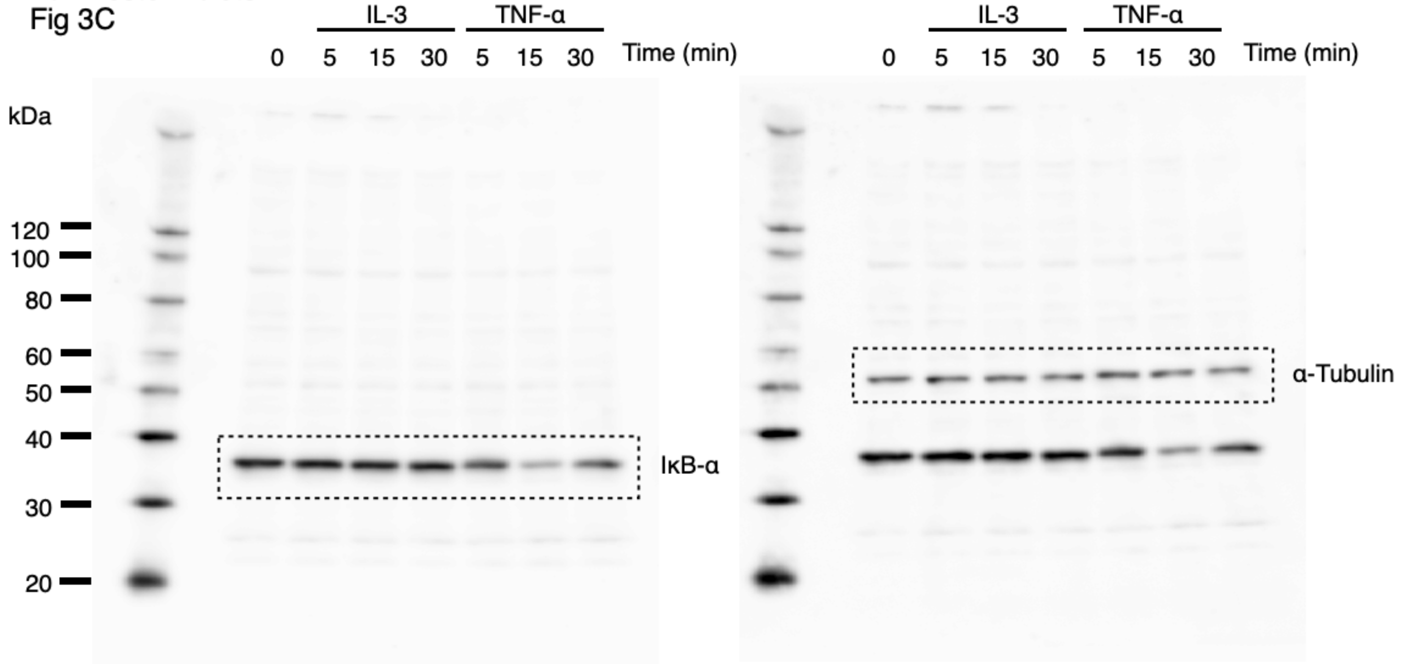

Full western blots
Fig 3D

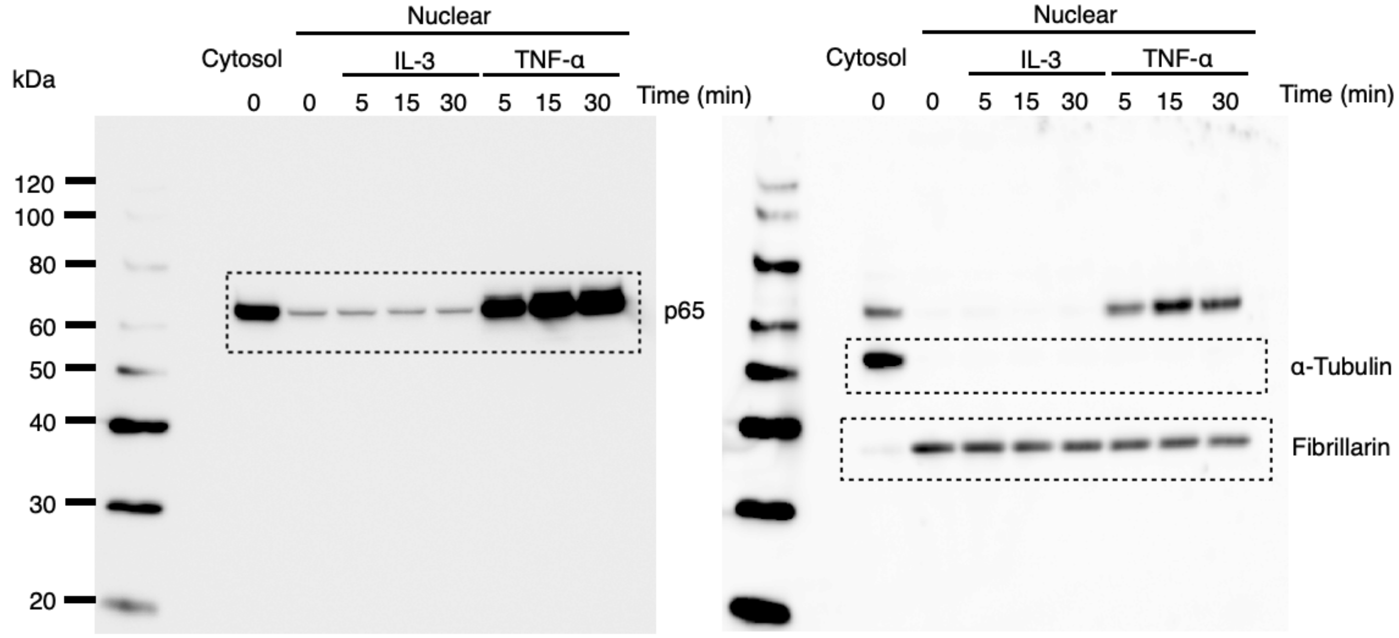

Full western blots
Fig 4A

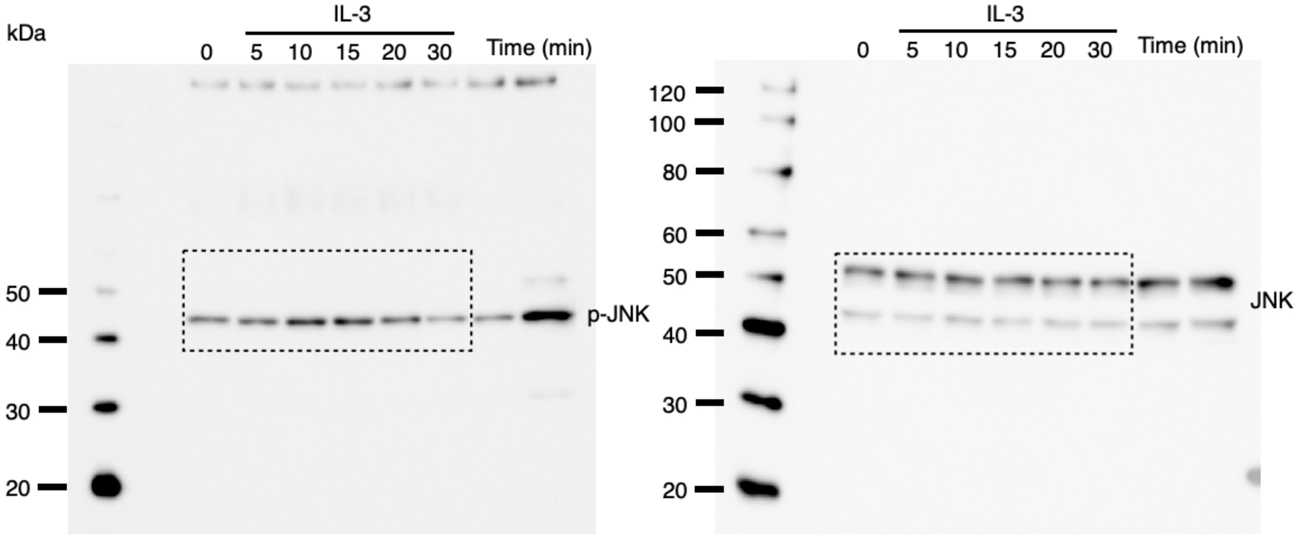

Full western blots
Fig 4B

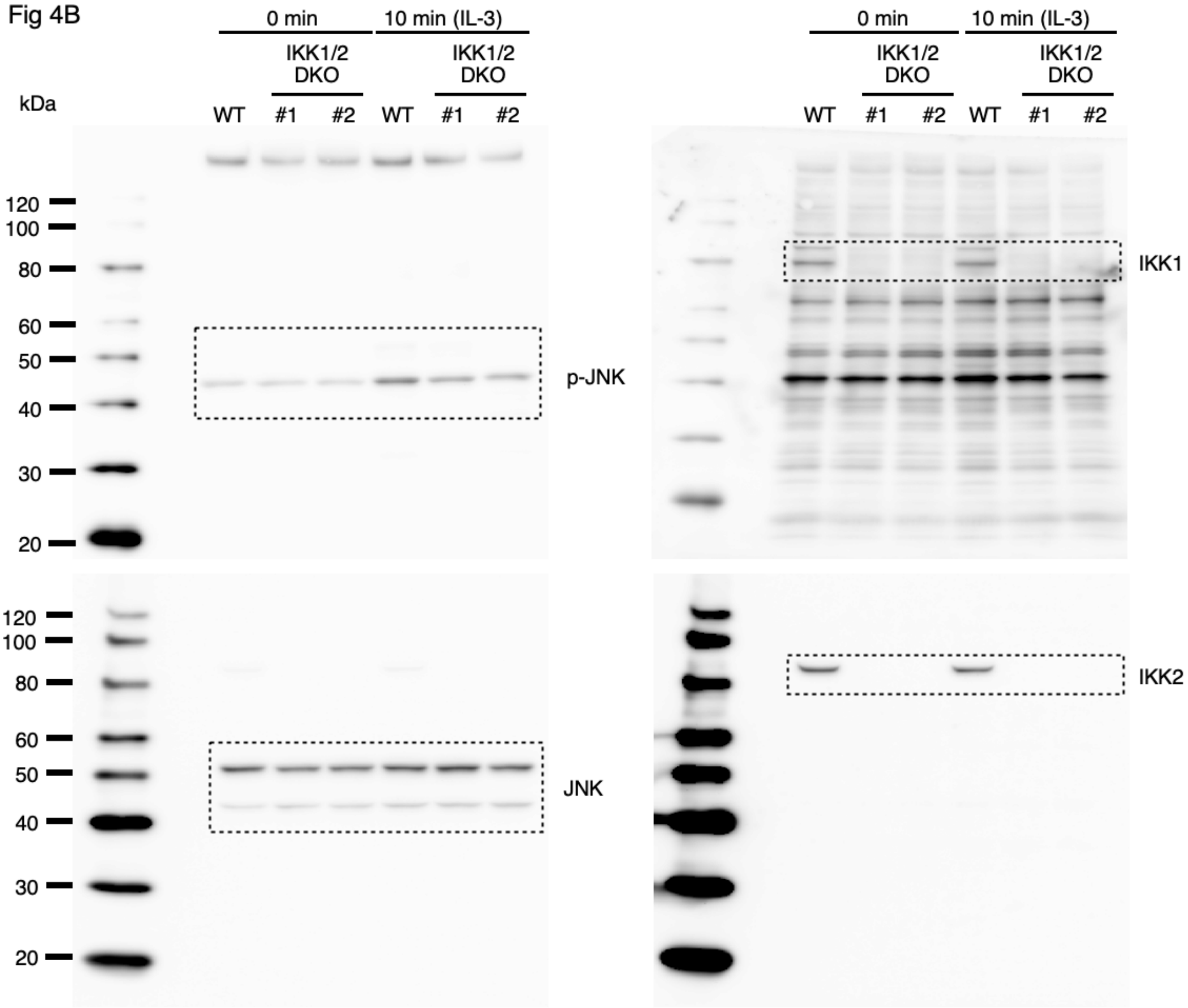

Full western blots
Fig 4C, D

Fig. S7 (continued)

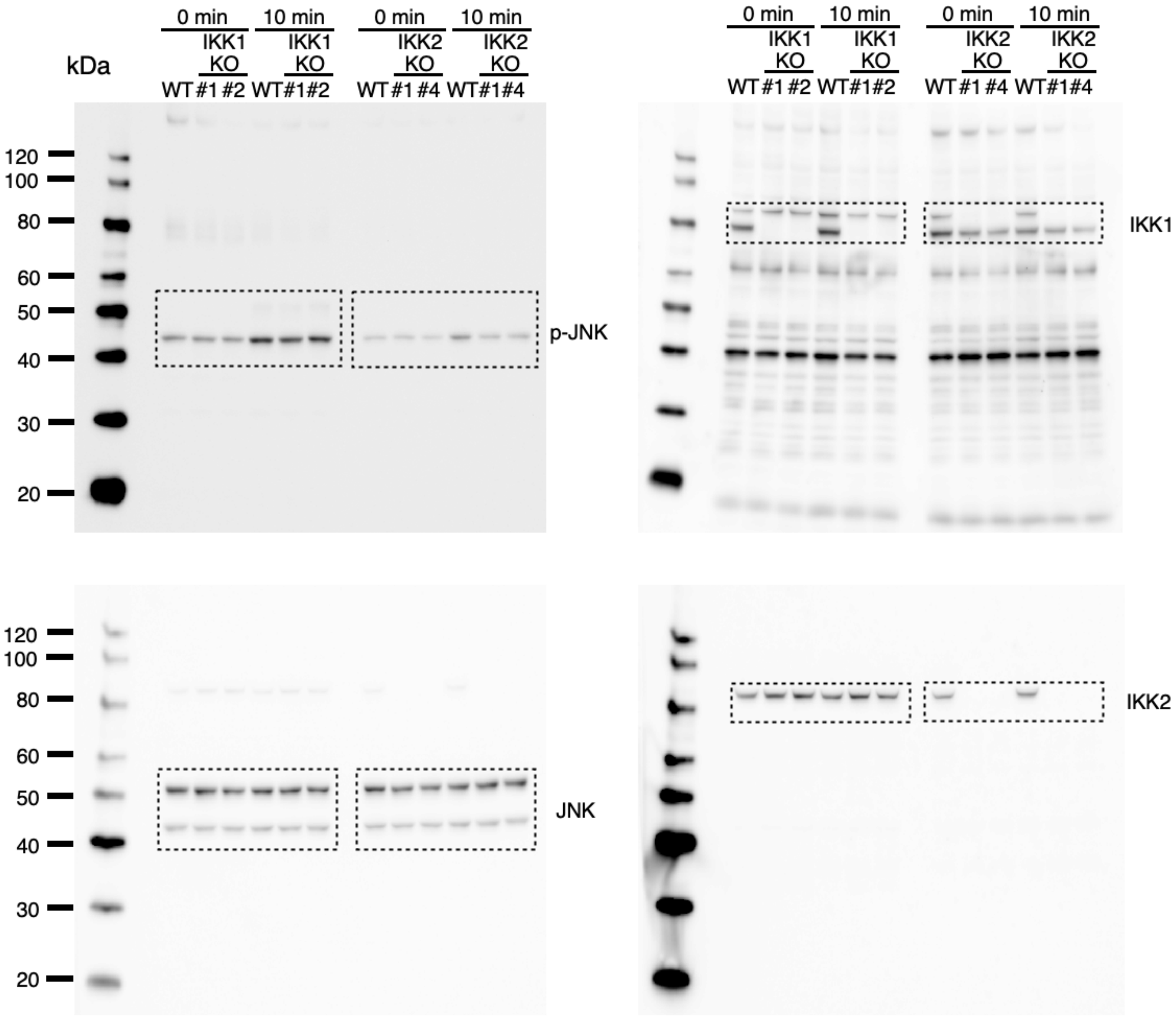

Full western blots
Figure S2A

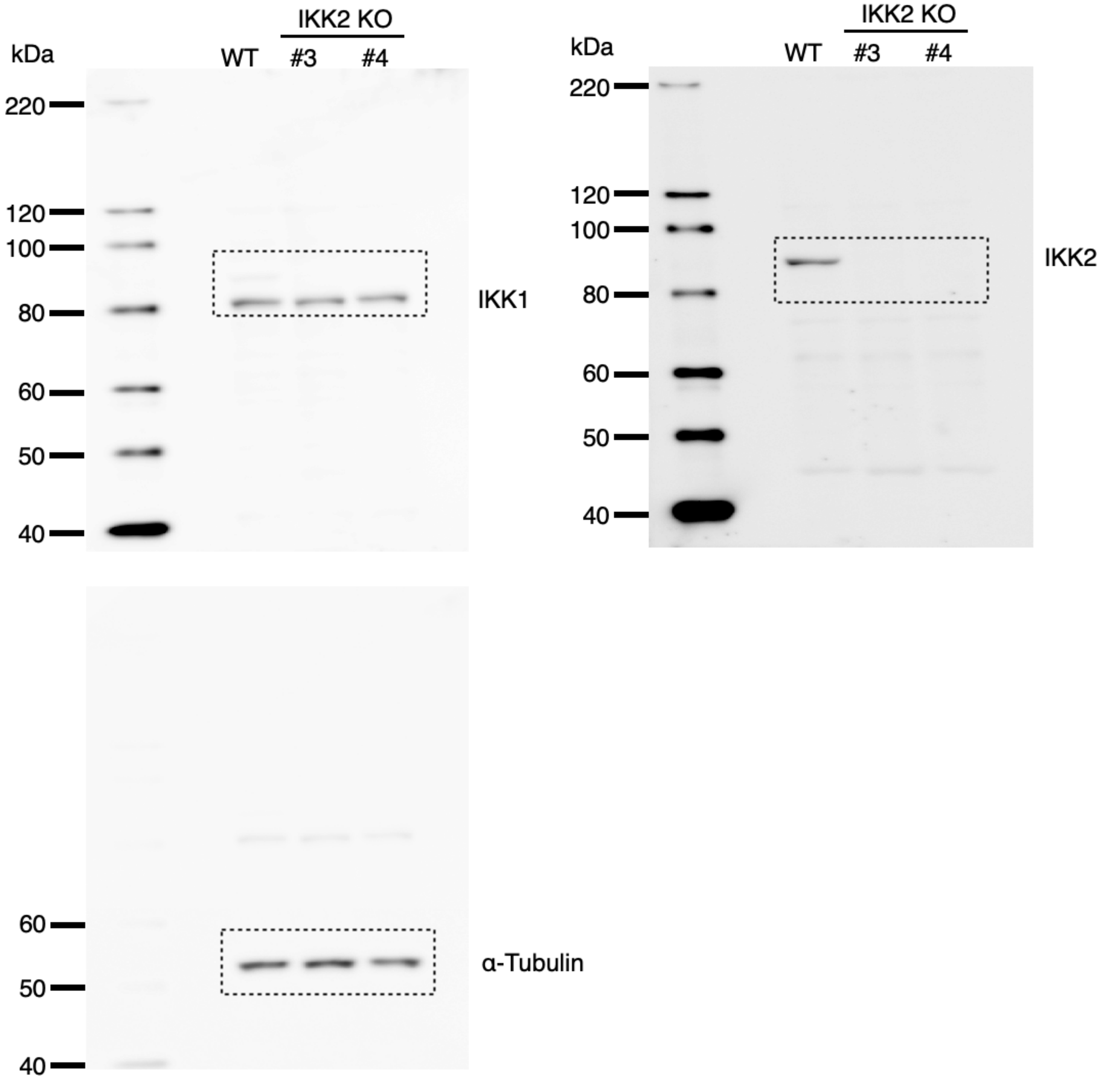

Full western blots
Figure S4A

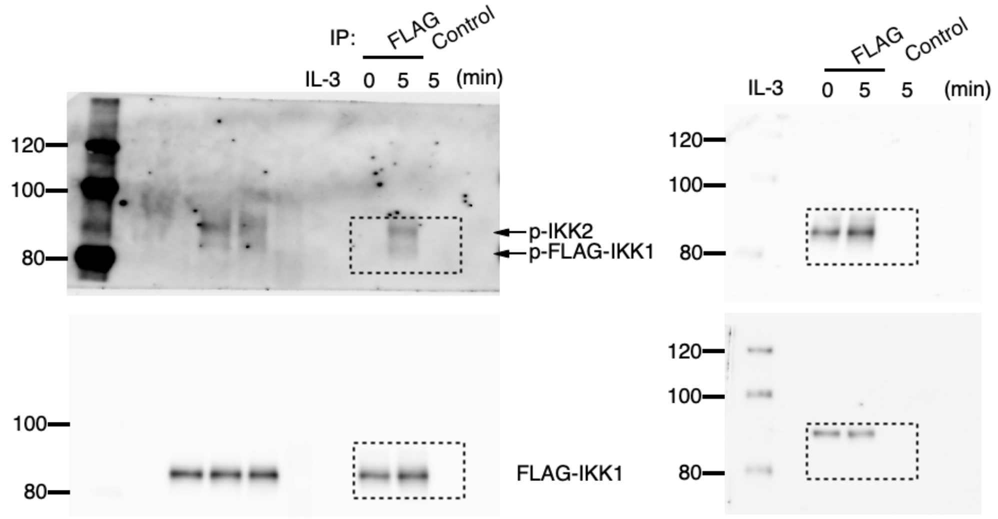

Full western blots
Figure S4B

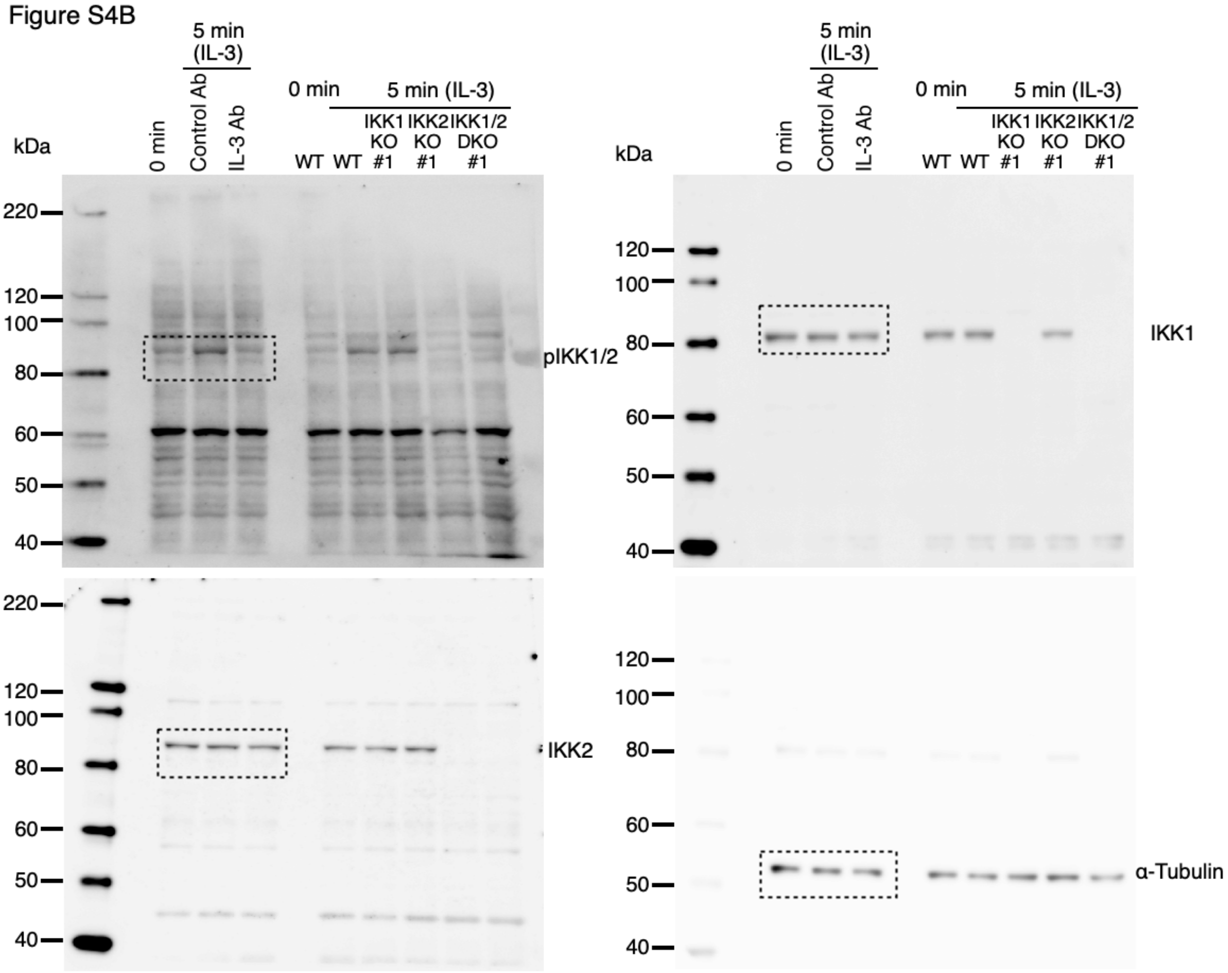

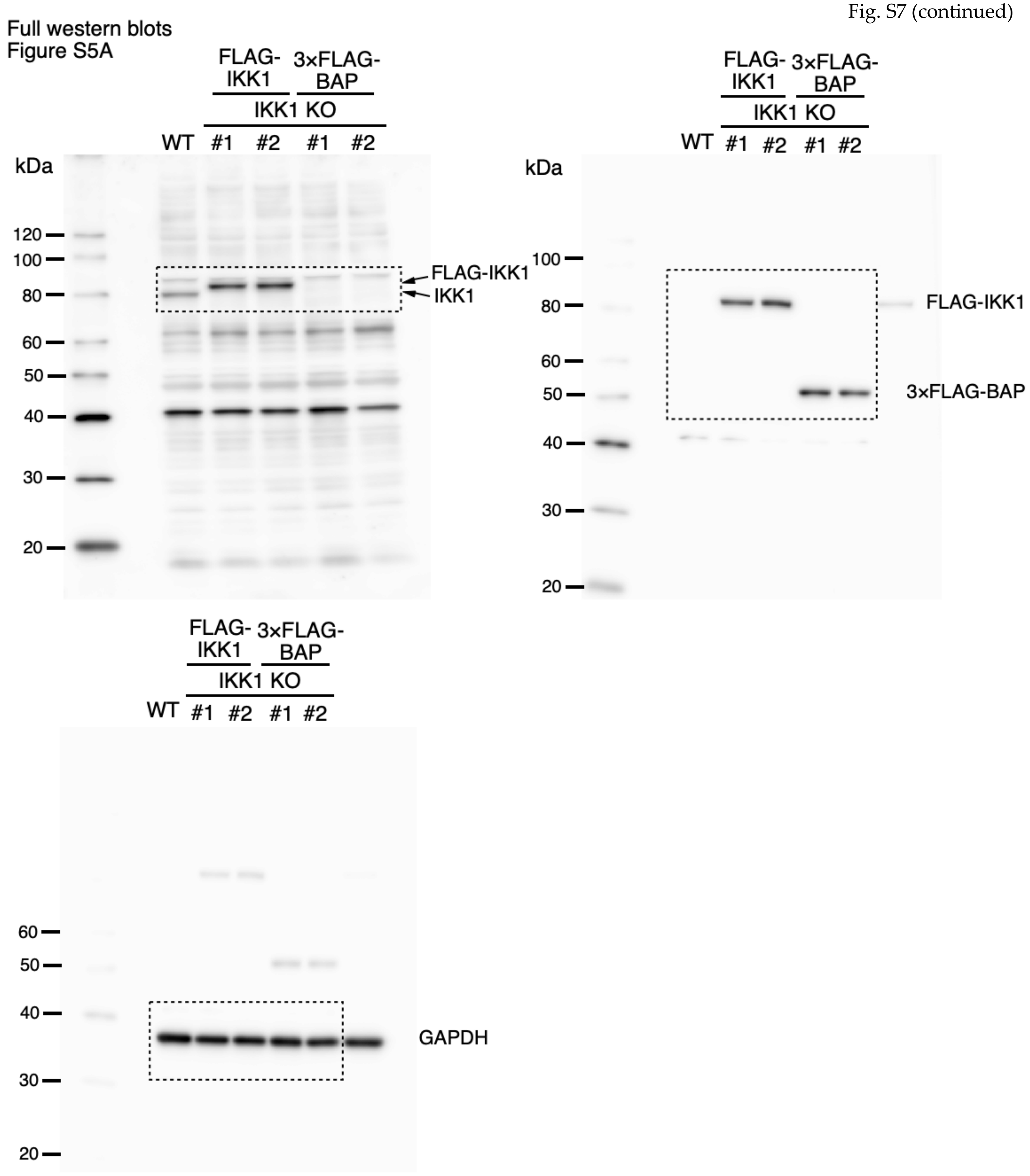

Figure S7. Full immunoblot images for the cropped images presented in the main and supplementary figures. Dotted squares indicate the regions presented in the figures.
